# Continuous evolution of Fermi arcs in a minimal ideal photonic Weyl medium

**DOI:** 10.1038/s41377-024-01632-w

**Published:** 2024-09-27

**Authors:** Yachao Liu, Mingwei Wang, Yongqing Huang, Guo Ping Wang, Shuang Zhang

**Affiliations:** 1 State Key Laboratory of Radio Frequency Heterogeneous Integration, College of Electronics and Information Engineering, Shenzhen University, Shenzhen, 518060 China; 2 Institute of Microscale Optoelectronics, Shenzhen University, Shenzhen, 518060 China; 3 Department of Physics, University of Hong Kong, Hong Kong, China; 4 Department of Electrical & Electronic Engineering, University of Hong Kong, Hong Kong, China

**Keywords:** Metamaterials, Nanophotonics and plasmonics

## Abstract

Propagation properties of electromagnetic waves in an optical medium are mainly determined by the contour of equal-frequency states in ${\boldsymbol{k}}$-space. In photonic Weyl media, the topological surface waves lead to a unique open arc of the equal-frequency contour, called the Fermi arc. However, for most realistic Weyl systems, the shape of Fermi arcs is fixed due to the constant impedance of the surrounding medium, making it difficult to manipulate the surface wave. Here we demonstrate that by adjusting the thickness of the air layer sandwiched between two photonic Weyl media, the shape of the Fermi arc can be continuously changed from convex to concave. Moreover, we show that the concave Fermi-arc waves can be used to achieve topologically protected electromagnetic pulling forces over a broad range of angles in the air layer. Our finding offers a generally applicable strategy to shape the Fermi arc in photonic Weyl media.

## Abstract (web summary)

The continuous evolution of the shape of the surface Fermi arc induces an electromagnetic pulling force that operates over a broad range of angles, effectively attracting a variety of small particles.

## Graphical abstract

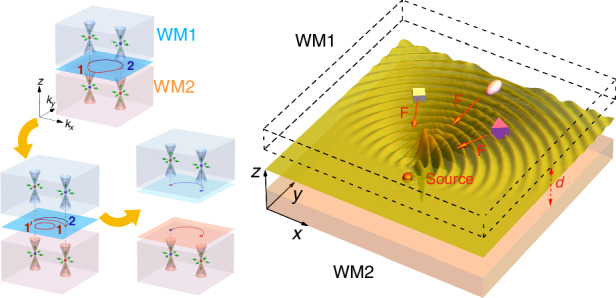

## Introduction

Fermi arcs, which connect two Weyl nodes (WNs) with opposite chirality, are highly indicative of the nontrivial bulk topology of Weyl media (WMs), thanks to the principle of bulk-boundary correspondence^1–7^. Moreover, Fermi arcs also offer insights into a diverse range of other physics related to the surface and bulk properties of WMs. For instance, in electronics, both the Fermi arc surface bands and bulk bands contribute to the conductivity of Weyl electrons, as evidenced in recent studies on Weyl-orbit quantum oscillations^8–12^. In classical wave systems, the shape of Fermi arcs determines the propagation properties of topologically protected surface waves, leading to phenomena such as negative reflection in acoustic systems and the realization of negative-index flat lenses in photonic systems^13–15^. Furthermore, Fermi arcs also play a role in changes to the beam wavefront and spatial trajectory upon reflection of waves in WMs^16^.

The imposition of boundary conditions on the surface terminations of a bulk WM has a significant impact on the geometry and connectivity of Fermi arcs. Notably, recent observations of Fermi arcs on the boundaries of an ideal photonic Weyl metamaterial, specifically on perfect electric conductor and perfect magnetic conductor boundaries, have revealed that they can effectively mimic positive and negative-index media, respectively^14^. Undoubtedly, the ability to arbitrarily tune the geometry and connectivity of Fermi arcs would be highly valuable for manipulating topological waves. While efforts have been made in condensed matter physics to achieve this, such as through the use of twisted interfaces constructed from different Weyl semimetals^17–19^, there is currently no established scheme for continuous tuning of Fermi arcs in photonics.

Here, we propose an approach to continuously tune the geometry of Fermi arcs in photonic Weyl metamaterials. By adjusting the thickness of an air layer between two photonic WMs, we demonstrate that Fermi arcs can transition from concave to convex open contours. Specifically, we assume that the WNs enclosed in these two WMs have coinciding momentum space positions, but opposite chirality. To highlight the potential of this approach, we investigate the electromagnetic force exerted by the Fermi arc waves on small objects. Remarkably, we observe a highly robust wide-angle electromagnetic pulling force when the linear momentum of the Fermi arc waves changes from positive to negative.

## Results

### Continuous evolution of Fermi arcs

**Fig. 1:**
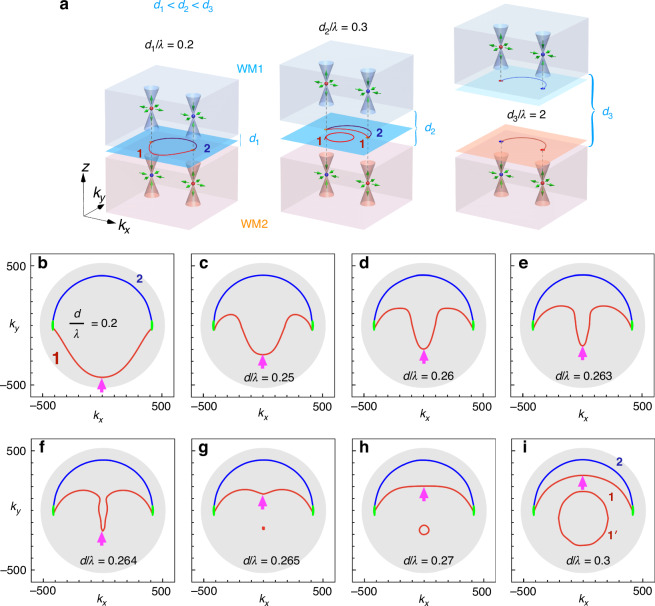
Continuous evolution of Fermi arcs in the minimal ideal photonic Weyl system. **a** Schematic showing how Fermi arcs can be adjusted by varying the thickness of an air layer (${d}_{1} \,<\, {d}_{2}\, < \,{d}_{3}$) sandwiched between two WMs (WM1 and WM2). These WMs have only two equal-frequency Weyl degenerates (red and blue dots) in momentum space, known as the minimal ideal Weyl system. The chirality of the Weyl degenerates in the upper and lower WMs is reversed, indicated by the different colors. The red and blue curves represent the two Fermi arcs (mode 1 and mode 2, respectively) that connect the projections of the Weyl degenerates. **b**–**i** Calculated Fermi arcs (denoted by colored curves) are shown for different air layer thicknesses $d$ (where $d=n\lambda$ is assumed in these calculations). Here, $\lambda$ is the vacuum wavelength at the operating frequency ($25{GHz}$). The green disks represent the projections of bulk states (WNs), while the gray background shows the corresponding light cone. The magenta arrows show the position of the wavevectors plotted in Fig. 3b

The configuration for achieving the continuous evolution of Fermi arcs is illustrated in Fig. 1a, where two WMs (WM1 and WM2) are separated by an air layer with thickness $d$. In this model, we assume the simplest Weyl system with only a pair of equal-frequency Weyl points (known as the minimal ideal Weyl system^20^) under broken time-reversal symmetry (TRS). The positions of the WNs in the upper and lower WMs are chosen to coincide in momentum space, as shown in Fig. 1a, with opposite chirality. When the two WMs are well-separated, the Fermi arcs on the lower surface of the upper WM (WM1) and the upper surface of the lower WM (WM2) are expected to have the same geometry (right panel). However, as the air layer thickness decreases, the coupling between the upper and lower surface waves becomes stronger, and the new Fermi arcs are more likely to gradually change (left and middle panels).

To analyze this modulation, we propose an effective medium model to describe the assumed WMs. In this model, the effective permeability is set as $\mu =1$, and the effective permittivity tensor is given by:

1 $$\mathop{\epsilon}\limits^{=}={\epsilon }_{0}\left[\begin{array}{ccc}{\epsilon }_{c}-{\epsilon }_{m}({\boldsymbol{k}}) & 0 & 0\\ 0 & {\epsilon }_{c}-{\epsilon }_{m}({\boldsymbol{k}})-{\epsilon }_{s} & -i\,{\epsilon }_{d}\\ 0 & i\,{\epsilon }_{d} & {\epsilon }_{c}-{\epsilon }_{s}\end{array}\right]$$

We introduce optical nonlocal response into the model through the ${\boldsymbol{k}}$-dependent “hydrodynamic” term ${\epsilon }_{m}\left({\boldsymbol{k}}\right)={\epsilon }_{c}{\omega }_{q}^{2}/[{\omega }^{2}-{\omega }_{0}^{2}+\alpha \left({k}_{x}^{2}+{k}_{y}^{2}\right)]$, where $\alpha$ is a constant accounting for the nonlocal effect, ${\epsilon }_{c}$ is the effective relative permittivity, and ${\omega }_{0}$ and ${\omega }_{q}$ are the effective resonance angular frequency^21^. The nonlocal effect plays a crucial role in our model as it determines the positioning of Weyl points within the Brillouin zone and whether they belong to type-I or type-II. The presence of type-I WNs facilitates the observation of distinct and well-defined Fermi arcs, making them more easily detectable^21^. Furthermore, to break the TRS and induce the emergence of Weyl degeneracies, we implement the gyro-electric response terms in the permittivity, ${\epsilon }_{s}={\omega }_{p}^{2}/({\omega }^{2}-{\omega }_{1}^{2})$ and ${\epsilon }_{d}={\omega }_{1}{\omega }_{p}^{2}/[\omega ({\omega }^{2}-{\omega }_{1}^{2})]$, where ${\omega }_{p}$ and ${\omega }_{1}$ are the effective plasma and cyclotron frequencies, respectively^22^. Notably, the sign of ${\epsilon }_{d}$ is opposite for the upper and lower WMs (WM1 and WM2), as it determines the chirality of the corresponding WNs. To achieve the desired sign of this parameter in practical experiments, we can either change the direction of the external magnetic field or incorporate magnetic materials with varying sequences into the metamaterial. More details, including the calculated 3D band structure and relevant parameters, can be found in the Supplementary information (SI), Sec. I and Fig. S1.

Afterwards, the Fermi arcs are plotted in the surface Brillouin zone to demonstrate their evolution with varying air layer thickness, as shown in Fig. 1b–i. Two distinct interface modes, labeled mode 1 and mode 2, are clearly observed and represented by the red and blue solid curves, respectively. These Fermi arcs satisfy the phase integral condition $2{\phi }_{1,2}+2{k}_{z}d=2\pi$, where ${\phi }_{1,2}$ is the reflection phase of the two distinct modes, the second term represents the round-trip propagation phase in the air layer, and the factor of two in front of ${\phi }_{1,2}$ accounts for the reflections caused by the upper and lower Weyl boundaries. A schematic of this phase integral condition is provided in the SI Fig. S2. To calculate the reflection phase of the eigenmodes (${\phi }_{1,2}$), we construct the reflection matrix of the WM capped with an air layer (see Materials and Methods). It is evident from these figures that the Fermi arcs of mode 1 change significantly with the air layer thickness, while that of mode 2 remains nearly unchanged. Interestingly, as the air layer thickness increases, the Fermi arcs of mode 1 change shape from concave to convex. This is also confirmed by Fourier transformations of the interface waves simulated from full-wave calculations, as shown in the SI Fig. S3. Additional states (red circles) demonstrated in Fig. 1g–i correspond to waveguide modes in this Weyl-air-Weyl (WAW) structure with a thicker air layer. Further simulation details are given in the SI Sec. II. Additionally, the simulated field distributions of mode 1 and mode 2 are included in the SI Figs. S4 and S5, respectively.

**Fig. 2:**
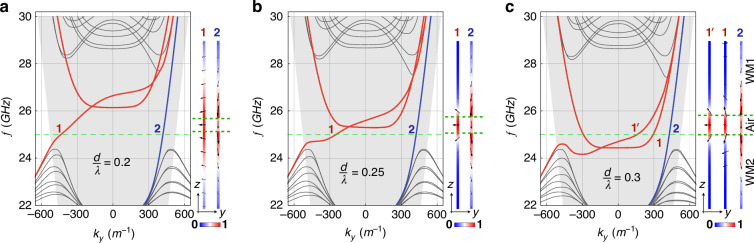
Band structures are plotted along the ${k}_{y}$ direction to show the changes in group velocities of the different Fermi arc modes. The red and blue curves, representing mode 1 and mode 2, respectively, are shown for three different air layer thicknesses: $d=0.2{\rm{\lambda }}$, $0.25{\rm{\lambda }}$, and $0.3{\rm{\lambda }}$ in **a**–**c**. The green dashed line represents the operating frequency at $25{GHz}$, with the gray background showing the corresponding light cone. The right insets present the field intensity distribution and electric field vectors of the representative modes (as marked in the band structures). **a**, **b** Negative group velocities are observed at the operating frequency for mode 1, as it has a negative wavevector ($2\pi f/{k}_{y}\,<\,0$) and positive slope ($2\pi \partial f/\partial {k}_{y} \,>\,0$). In contrast, mode 2 has a positive group velocity ($2\pi f/{k}_{y} \,> \,0$, $2\pi \partial f/\partial {k}_{y}\, > \,0$). **c** Both Fermi arc mode 1 and mode 2 have positive group velocities

To further demonstrate the modulation of the Fermi arc interface states by varying the air layer thickness, we present the band structures of the WAW structure in Fig. 2. Three different thicknesses, $d=0.2{\rm{\lambda }}$, $0.25{\rm{\lambda }}$, and $0.3{\rm{\lambda }}$ (where ${\rm{\lambda }}$ is the operating wavelength at $25{GHz}$ in our calculations) are plotted. As in Fig. 1b–i, the bands of the two interface modes are colored in red and blue, respectively. In these plots, the bands of both interface modes exhibit a shift towards the positive ${k}_{y}$ direction with increasing $f$, indicating a positive group velocity of $2\pi \partial f/\partial {k}_{y}\, > \,0$ along the $y$ direction. However, for small thicknesses ($d=0.2{\rm{\lambda }}$, $0.25{\rm{\lambda }}$), a negative phase velocity ($2\pi f/{k}_{y}\, < \,0$) is obtained, corresponding to the concave-shaped Fermi arcs. This suggests that the concave Fermi arc waves carry negative linear momentum (opposite to the group velocity), as is the case in Fig. 1b–f. In addition, we plot the field intensity distribution and electric field vector diagrams corresponding to different modes on the right side of each band structure. It is observed that for mode 1, the field intensity is mainly concentrated within the air layer, with horizontal electric field polarization at the middle interface. In contrast, for mode 2, the field intensity is concentrated within the Weyl materials on both sides, exhibiting vertical electric field polarization. Thus, the coupling effect introduced within the air layer has a significant impact on mode 1. Mode ${1}^{{\prime} }$ in Fig. 2c represents an additional state that emerges as the air layer thickens, corresponding to the closed curves in Fig. 1g–i, with field intensity and electric field vector distributions similar to those of mode 1. Further details on the simulation of the band structures can be found in the SI Sec. III.

### Electromagnetic force exerted by the Fermi arc waves

**Fig. 3:**
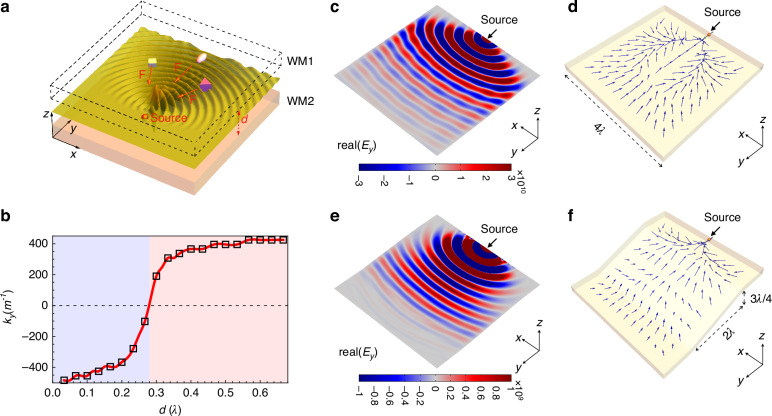
Electromagnetic pulling force achieved by utilizing the concave Fermi arc waves. **a** Schematic of the configuration used to support the pulling waves. An electric dipole (y direction) is enclosed in the air layer, which serves as the source of waves. Scatterers in various shapes are attracted by the Fermi arc waves. **b** Variation of the negative most wavevector $[\min ({k}_{y})$, marked as magenta arrows in Fig. 1b–i] is present along with the thickness of the air layer. **c**, **d** Distributions of the electric field and force vectors are plotted, respectively, for a planar air layer with thickness $d=0.25\lambda$. The force vectors show the direction and magnitude of the electromagnetic force exerted on a spherical dielectric ball with radius $\lambda /20$ and permittivity $\epsilon =4$. **e**, **f** Similar to **c**, **d** but for a sloping air layer. Pulling force is achieved in both cases as the calculated force vectors point toward the wave source

We now have demonstrated that the Fermi arcs can be manipulated by adjusting the thickness of the air layer. To further illustrate the potential of this approach, we investigate the electromagnetic force exerted by these Fermi arc waves. In Fig. 3a, we present a schematic representation of our analysis, where small objects of various shapes are utilized to evaluate the electromagnetic force. Unlike boundary waves that typically occur at interfaces between different topological media, the presence of the air layer in our model creates an empty gap that allows small objects to pass through, potentially enabling a transport channel for specific particles.

A qualitative evaluation of the momentum imparted to the objects can be formulated as

2 $$\Delta {P}_{y}={P}_{y}^{i}-{{P}_{y}}^{s}=\Delta N\,{{\hslash}}\,({k}_{y}^{i}-{k}_{y}^{s})$$

where $\Delta N \,> \,0$ is the number of scattered photons, ${k}_{y}^{i}$ and ${k}_{y}^{s}$ are the incident and scattered wavevectors respectively, and ${\boldsymbol{\hslash }}$ is the reduced Planck constant. It is worth noting that ${k}_{y}^{i}$ and ${k}_{y}^{s}$ can both be negative when the Fermi arc is concave (${k}_{y} \,< \,0$), implying that the phase velocity of the incident and scattered waves are both opposite to the direction of their group velocities. To achieve a negative $\Delta {P}_{y}$ in our model, we selectively excite the negative most ${k}_{y}$ states that satisfy $\left|{k}_{y}^{i}\right| > \left|{k}_{y}^{s}\right|$. In the examples shown in Fig. 1b–i, the positions of the negative most ${k}_{y}$ states (${k}_{x}=0$) are indicated by the magenta arrows. We plot the variation of this negative most ${k}_{y}$ state with respect to the air layer thickness in Fig. 3b, which reveals negative values in the range $0\, < \,d \,< \,0.28\lambda$. Using the equation $F=\Delta P{{/}}{{\Delta }}t$, we can obtain an electromagnetic pulling force ($F \,< \,0$) in this region since the time variation ${\boldsymbol{\triangle }}t$ is always positive. A schematic of this momentum conservation law is provided in the SI Fig. S6. Notably, our findings present a novel mechanism for achieving an electromagnetic pulling force, which has been the subject of extensive efforts in the past few decades^23–28^.

Furthermore, unlike the previously reported schemes that rely on topologically protected waves to achieve the electromagnetic pulling force^29–31^, our model does not involve a mode-conversion process, as proved by the scattering fields shown in the SI Fig. S7. It is worth noting that the inclusion of the mode-conversion process can actually decrease the performance of a pulling wave in the presence of multiple small objects (more than one) simultaneously. This is because the mode-conversion process can be reversed due to multiple scattering, which hinders the pulling of small objects. In contrast, our approach presents a potential solution to this challenge, as evidenced by the comparison of different optical pulling schemes shown in the SI Fig. S8.

### Robust wide-angle electromagnetic pulling forces

To visualize the electromagnetic pulling force directly, we utilize the Maxwell stress tensor integrated along a closed surface that surrounds the objects, denoted as 〈***F***〉 = *∫*〈***M***〉*dS*, where 〈***M***〉 is defined as follows:

3 $$\langle {\boldsymbol{M}}\rangle =\frac{1}{2}\,{\mathrm{Re}}[{\boldsymbol{D}}\otimes {{\boldsymbol{E}}}^{* }+{\boldsymbol{H}}\otimes {{\boldsymbol{B}}}^{* }-\frac{1}{2}\mathop{I}\limits^{\leftrightarrow}({\boldsymbol{E}}\,\cdot\, {{\boldsymbol{D}}}^{* }+{\boldsymbol{H}}\,\cdot\, {{\boldsymbol{B}}}^{* })]$$

Here, $\langle \,\cdot\,\rangle$ represents the time average, ${\boldsymbol{\otimes }}$ denotes the dyadic operation, and $\mathop{I}\limits^{\leftrightarrow}$ is the unit tensor. We note that the Minkowski momentum rather than Abraham momentum is applied, as it has been proven that the momentum associated with the matter (Minkowski momentum) contributes to the electromagnetic force in an immersed system, as is the case in our study^32^.

We present the electric field distribution [${real}({E}_{y})$] across the middle section of the planar air gap in Fig. 3c, along with the corresponding force vectors shown in Fig. 3d. To perform these calculations, we consider a spherical object with a radius of ${r}_{b}=\lambda /20$ and a relative permittivity of $\epsilon =4$ positioned in an air gap of width $d=0.25\lambda$. The closed surface selected for integration is the surrounding surface with a scaled radius of $1.1\times {r}_{b}$. As shown Fig. 3d, the force vectors are all directed towards the wave source, providing unambiguous evidence of pulling forces in our model. See the electric field distributions of the other two components (${E}_{x}$ and ${E}_{z}$) in the SI Fig. S9. Additionally, we investigate a sloping air layer sandwiched between the WMs, as shown in Fig. 3e, f. Remarkably, the interface waves are able to circumvent the slope defects, and as a result, the electromagnetic pulling force is well preserved even after passing through the slope defect, as demonstrated in Fig. 3f. To further demonstrate the topological protection characteristics of these concave Fermi arc waves, we introduced another cylindrical obstacle made of perfect electric conductor (PEC) in the air layer and calculated the corresponding optical force vector distribution. See Fig. S10 in SI for the corresponding results. Here, we note that the topologically nontrivial characteristics of the photonic Weyl points are the underlying reason for the Fermi arc waves’ ability to circumvent the slope defects and the cylindrical obstacles.

**Fig. 4:**
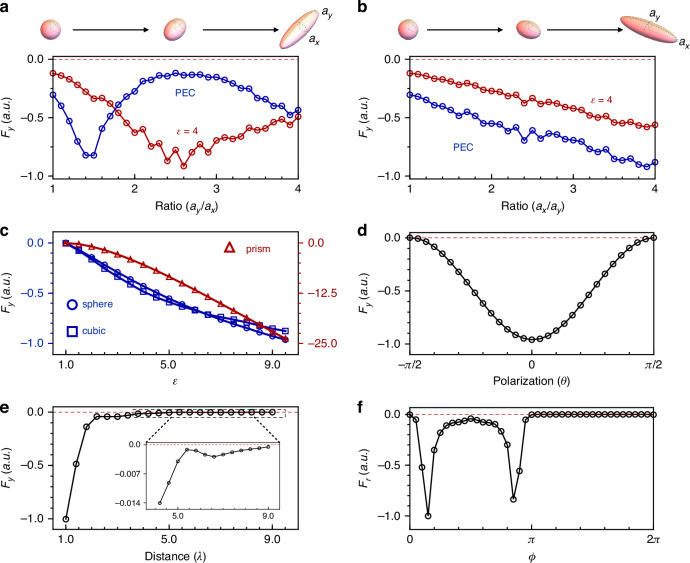
Robust wide-angle electromagnetic pulling forces. **a**, **b** Magnitude of the electromagnetic force acting on ellipsoidal scatterers (see the top insets) with different major-to-minor axis ratios *(*${a}_{y}/{a}_{x}$, or ${a}_{x}/{a}_{y}$), where the minor axis is consistently set at $\lambda /10$ for simplicity. The scatterers are assumed to be made of materials with a permittivity of $\epsilon =4$ (red curves) or to be PEC metal (blue curves). The distance between the scatterers and source dipole is kept at $2.8\lambda$. **c** Scatterers in the shape of a sphere (radius $\lambda /10$), cube (side length $\lambda /5$), and triangle prism (side length $\lambda /5$) with different permittivity ($\epsilon$) are investigated. The distances between the scatterers and the source dipole remain constant at $2\lambda$. **d** Forces varying with the polarization of the source dipole [${\hat{e}}_{y}\cos \left({\rm{\theta }}\right)+{\hat{e}}_{z}\sin \left({\rm{\theta }}\right)$], where a sph**e**rical scatterer ($r=\lambda /10$, $\epsilon =4$) at a fixed distance ($2.8\lambda$) from the source is examined. **e**, **f** Force variations in response to changes in the distance to the point source and the azimuthal angle $\phi$ (an angle around the point source, rotating away from the $+x$ axis in the *x*-*y* plane), respectively. A spherical scatterer with $r=\lambda /10$, $\epsilon =4$ is studied. The thickness of the air layer is maintained at $d=0.25\lambda$ for all the calculations presented in this figure

The utilization of topologically protected Fermi arc waves ensures the robustness of the electromagnetic pulling force against variations in geometric and material parameters of scatterers. This is demonstrated by evaluating the electromagnetic force (${F}_{y}$) of a dielectric ($\epsilon =4$)/PEC scatterer in the shape of an ellipsoid with varying major-to-minor axis ratios. As shown in Fig. 4a, b, the results consistently show a pulling force (${F}_{y}\, < \,0$). Further investigations involve changing the shape of scatterers from a sphere to a cube or prism and varying the permittivity from $\epsilon =\,1$ to $9$. In all cases, the pulling force is preserved, as illustrated in Fig. 4c.

The electromagnetic force generated by the Fermi arc waves, including mode 1 and mode 2, is also expected to be controlled by changing the polarization of the wave source, as the direction of the source dipole can be chosen to approximately select these modes {See details in the SI Figs. S4 and S5}. To demonstrate this modulation, we varied the direction of the source dipole in the y-z plane defined by the angle with respect to the y-axis, denoted as $\theta$, and measured the resulting electromagnetic force using a spherical scatterer with parameters of $r=\lambda /10$ and $\epsilon =4$, while keeping the distance to the source fixed at $2.8\lambda$. The dependence of the resulting force (${F}_{y}$) on the polarization angle $\theta$ is shown in Fig. 4d, revealing a modulated magnitude of the pulling force. Notably, even at $\theta =\pm \pi /2$ (z-axis), where mode 2 is predominantly excited, the force is still pulling. This is because the scattered field include not only the mode 2 states with positive linear momentum, but also the mode 1 states with negative linear momentum in these cases. Further information about the scattered fields can be found in the SI Fig. S11.

The dependence of the electromagnetic force on the distance from the light source is presented in Fig. 4e, where a dielectric ball with a radius of $r=\lambda /10$ and a relative permittivity of $\epsilon =4$ is considered. As illustrated, the electromagnetic force always acts in the pulling direction (${F}_{y}\, < \,0$), and its magnitude decreases as the object moves away from the source. In addition, we examine the variation of the electromagnetic force with the azimuthal angle $\phi$ in Fig. 4f, with the distance between the object and the point source fixed at $2\lambda$. For $\pi\, < \,\phi \le 2\pi$, the electromagnetic force (${F}_{r}$) is zero as backward waves are not allowed. While for the other half plane, $0 \,< \,\phi \le \pi$, the pulling force is observed. The maximum pulling force is observed at the directions $\phi =0.14\pi$ and $0.86\pi$, where the field strength is at its peak. See the field strength distributions in the SI Fig. S12. Here we note that the broad range of angles that support the pulling effect in our model is due to the wide range of *k* vectors provided by the Fermi arc waves. See the comparison between our proposal and a general scheme in the SI Fig. S13.

## Discussion

In conclusion, we have demonstrated the continuous evolution of Fermi arcs from a concave to a convex open contour in a sandwiched Weyl-air-Weyl structure, which offers a convenient way to adjust the linear momentum of a topologically protected wave from positive to negative. Using the Maxwell stress tensor, we have also analyzed the electromagnetic force generated by this Fermi arc wave on objects with different shapes and material parameters. The resulting electromagnetic force acts over a broad range of angles and can be easily modulated from a pulling to a pushing force, making it a robust “optical tweezer” for attracting different small objects. Drawing on experimental schemes reported in the recent literature on photonic Weyl materials, we have derived most of the main results in this work at microwave frequencies. Nonetheless, the conclusions regarding the continuous evolution of the Fermi arcs can be extended to higher frequencies, which is especially meaningful in light of the realization of photonic Weyl materials in the terahertz and near-infrared frequency ranges recently^33,34^. In the future, we intend to introduce magneto-optical materials, such as transparent ceramics, to break the system’s TRS and to extend our findings to the optical frequency range^35,36^. Moreover, the practical potential of our theoretical model lies in the recent realization of the minimal ideal photonic Weyl medium (with only two equal-frequency Weyl points) in various photonic systems^15,22,33^. We anticipate that the mechanism presented here will extend the application of topological waves to the field of optical manipulation.

## Materials and methods

### Reflection matrix of the Weyl-air interface

We first consider the interface between the Weyl medium (lower half space) and the air bulk (upper half space), which lies in the x-y plane. A wave coming from the air side is reflected at the interface, which can be described by using the boundary continuity of the tangential component of the electromagnetic waves. In the bulk of air, the two orthogonal eigen modes (TE and TM) can be expressed as $\{{E}_{1},{H}_{1}\}$ and $\{{E}_{2},{H}_{2}\}$. For a fixed transverse wavevector ${k}_{\perp }=\{{k}_{x},{k}_{y}\}$, the incoming and outgoing waves can be expanded by these two modes with the contrast longitudinal wavevector ${k}_{{\rm{z}}}\, > \,0$ ($+$) and ${k}_{{\rm{z}}}\, < \,0$ ($-$) correspondingly. In the bulk of Weyl medium, two orthogonal modes with the same transverse wavevector ${k}_{\perp }$ can be obtained by choosing ${Im}({k}_{{\rm{z}}})\, > \,0$, which indicates a decaying wave goes into the Weyl medium, and can be expressed as $\{{E}_{3},{H}_{3}\}$ and $\{{E}_{4},{H}_{4}\}$ respectively. Then, the boundary continuity described by the following equation should be satisfied:

$$\left[\begin{array}{cccc}{E}_{1x}^{-} & {E}_{2x}^{-} & {E}_{3x} & {E}_{4x}\\ {E}_{1y}^{-} & {E}_{2y}^{-} & {E}_{3y} & {E}_{4y}\\ {H}_{1x}^{-} & {H}_{2x}^{-} & {H}_{3x} & {H}_{4x}\\ {H}_{1y}^{-} & {H}_{2y}^{-} & {H}_{3y} & {H}_{4y}\end{array}\right]\left[\begin{array}{c}{I}_{a}\\ {I}_{b}\\ {O}_{a}\\ {O}_{b}\end{array}\right]=\left[\begin{array}{cc}{E}_{1x}^{+} & {E}_{2x}^{+}\\ {E}_{1y}^{+} & {E}_{2y}^{+}\\ {H}_{1x}^{+} & {H}_{2x}^{+}\\ {H}_{1y}^{+} & {H}_{2y}^{+}\end{array}\right]\left[\begin{array}{c}{R}_{a}\\ {R}_{b}\end{array}\right]$$

where the superscript $+/-$ represents the incoming and outgoing wave in the air layer, the letters $I$, $R$, $O$ represent the magnitudes of incoming, reflecting, and decaying waves, respectively. By solving the equation, a reflection matrix is written as

$$\left[\begin{array}{c}{R}_{a}\\ {R}_{b}\end{array}\right]=\left[\begin{array}{cc}{raa} & {rab}\\ {rba} & {rbb}\end{array}\right]\left[\begin{array}{c}{I}_{a}\\ {I}_{b}\end{array}\right]$$

Eigen modes of this reflection matrix represent a state for which its polarization will not be changed after the reflection. The phase change of the two eigen modes can thus be derived as:

$${\phi }_{1,2}={Arg}({r}_{1,2})$$

where ${r}_{\mathrm{1,2}}$ is the eigen value of the reflection matrix. We get the Fermi arcs in the main text by considering $2{\phi }_{1,2}+2{k}_{z}d=2\pi$, which is due to the fact that the reflection phase of the upper and lower Weyl-air interfaces in our WAW structure and the round-trip propagation phase in the air layer should both be considered in the interplaying of the interface waves.

## Supplementary information

Supplemental material for Continuous evolution of Fermi arcs in a minimal ideal photonic Weyl medium
