# Supplementary material for: Continuous evolution of Fermi arcs in a minimal ideal photonic Weyl medium
Source: Light Sci Appl. 2024 Sep 27;13:276. doi: 10.1038/s41377-024-01632-w (PMC11427711; doi:10.1038/s41377-024-01632-w)
Supplement: Supplementary file 1 — Supplemental material for Continuous evolution of Fermi arcs in a minimal ideal photonic Weyl medium [file 41377_2024_1632_MOESM1_ESM.docx]

**Supplementary Information for**

**Continuous evolution of Fermi arcs in a minimal ideal photonic Weyl medium**

Yachao Liu^1#^, Mingwei Wang^1,2^, Yongqing Huang^1,2^, Guo Ping Wang^1†^, and Shuang Zhang^3,4*^

1. *State Key Laboratory of Radio Frequency Heterogeneous Integration, College of Electronics and Information Engineering, Shenzhen University, Shenzhen 518060, China*
2. *Institute of Microscale Optoelectronics, Shenzhen University, Shenzhen 518060, China*
3. *Department of Physics, University of Hong Kong, Hong Kong, China*
4. *Department of Electrical & Electronic Engineering, University of Hong Kong, Hong Kong, China*

Email: Tel:

^#^Yachao Liu: [yachaoliu@szu.edu.cn](mailto:yachaoliu@szu.edu.cn) +86 13787115809

Mingwei Wang wangmingwei2022@email.szu.edu.cn

Yongqing Huang huangyongqing2022@email.szu.edu.cn

^†^Guo Ping Wang: [gpwang@szu.edu.cn](mailto:gpwang@szu.edu.cn) +86 13823556131

^*^Shuang Zhang: [shuzhang@hku.hk](mailto:shuzhang@hku.hk) +852 2859 7944

Note I. Band structure of the minimal ideal Weyl medium

Here, we recall the full form of the effective permittivity of the Weyl medium provided in the main text:

$\epsilon=\epsilon_{0}\left[ \begin{matrix} \epsilon_{c}-\frac{\epsilon_{c}\omega_{q}^{2}}{\omega^{2}-\omega_{0}^{2}+\alpha\left( k_{x}^{2}+k_{y}^{2} \right)} & 0 & 0 \\ 0 & \epsilon_{c}-\frac{\epsilon_{c}\omega_{q}^{2}}{\omega^{2}-\omega_{0}^{2}+\alpha\left( k_{x}^{2}+k_{y}^{2} \right)}-\frac{\omega_{p}^{2}}{\omega^{2}-\omega_{1}^{2}} & -i \frac{\omega_{1}\omega_{p}^{2}}{\omega(\omega^{2}-\omega_{1}^{2})} \\ 0 & i \frac{\omega_{1}\omega_{p}^{2}}{\omega(\omega^{2}-\omega_{1}^{2})} & \epsilon_{c}-\frac{\omega_{p}^{2}}{\omega^{2}-\omega_{1}^{2}} \end{matrix} \right]$.

The related parameters are chosen as $\epsilon_{c}=0.83$, $\alpha=6.8\times2\pi\times{10}^{15} {rad}^{2}/s^{2}$, $\omega_{q}=1.8\times2\pi\times{10}^{10} rad/s$, $\omega_{0}=2.22\times2\pi\times{10}^{10} rad/s$, $\omega_{p}=5\times{10}^{10} rad/s$, and $\omega_{1}=1.22\times{10}^{10} rad/s$ in the related calculations of this work. The Weyl frequency resulting from these parameters is $25.1GHz$, which falls within the microwave range, making it readily achievable in experiments.

To visualize the 3D band structure of the Weyl medium, we derive the Maxwell equation of the Weyl medium as

$\left( \left[ \begin{matrix} 0 & -K\times\\ K\times& 0 \end{matrix} \right]-\omega\left[ \begin{matrix} \epsilon& 0 \\ 0 & \mu\end{matrix} \right] \right)\left[ \begin{matrix} E \\ H \end{matrix} \right]=0$,

where $K\times$ represents the matrix $\left[ 0,-k_{z},k_{y};k_{z},0,-k_{x};-k_{y},k_{x},0 \right]$. The band structure is then obtained by calculating the determinant of the left matrix, as shown in Fig. S1.

Note II. Electromagnetic fields and optical force calculations in the Weyl medium

To numerically calculate the effective medium model provided in the main text, the wave equation of electric field is firstly examined in this work, which can be written as

$\nabla\times\left( \frac{1}{\mu}\nabla\times\boldsymbol{E} \right)-\epsilon\frac{\omega^{2}}{c^{2}}\boldsymbol{E}=0$,

where the analytic form of $\epsilon$ is given by the Eq.(1) in the main text. Because the nonlocal effect is considered in our model to fit the electromagnetic response of a practical Weyl metamaterial, higher order spatial derivatives are presented in the numerical calculations, which makes the above equation cannot be solved directly by adapting a general numerical program, such as the CST and COMSOL.

To avoid this problem, we introduce the method developed in our previous work [1] to reduce the order of the spatial derivatives in the wave equation by adding an auxiliary vector $\boldsymbol{J}=\left( J_{1},J_{2} \right)$, which finally makes the wave equation in the form (weak form) as

$\text{weak form=}(\nabla\times\boldsymbol{E})\cdot(\nabla\times\boldsymbol{T}\boldsymbol{E})+\boldsymbol{J}\cdot({TE}_{x},TE_{y})\boldsymbol{+(}\omega_{0}^{2}-\omega^{2})\boldsymbol{J}\cdot\boldsymbol{TJ-}\alpha(\partial_{x}+\partial_{y})\boldsymbol{J}\cdot(\partial_{x}+\partial_{y})\boldsymbol{TJ}-\epsilon_{0}\mu_{0}\omega^{2}[\epsilon_{c}\boldsymbol{E}\cdot\boldsymbol{TE}\mathbf{-}\epsilon_{s}\boldsymbol{(}E_{y},E_{z}\boldsymbol{)}\cdot\boldsymbol{(}TE_{y},TE_{z}\boldsymbol{)}\mathbf{-}i\epsilon_{d}\boldsymbol{(}E_{z},E_{y}\boldsymbol{)}\cdot\boldsymbol{(}TE_{y},TE_{z}\boldsymbol{)}\mathbf{-}\epsilon_{c}\omega_{q}^{2}(E_{x},E_{y})\cdot\boldsymbol{TJ}]$.

Here, $\boldsymbol{E}$ =($E_{x}$, $E_{y}$, $E_{z}$) is the electric field; $TX$ is the test function of corresponding field $X$, $TX=test(X)$; $i$ is the imaginary unit, $\epsilon_{0}$ and $\mu_{0}$ are the vacuum permittivity and permeability; $\omega$ is the angular frequency; ($\epsilon_{c},\epsilon_{d}, \epsilon_{s},\alpha, \omega_{q}, \omega_{0}, \omega_{p}, \omega_{1}$) are defined in Note I.

Then the other field components can be deduced from the electric field$\boldsymbol{E}$ and the auxiliary field $\boldsymbol{J}$. That is

$D_{x}=\epsilon_{0}\epsilon_{c}E_{x}-\frac{J_{1}}{\mu_{0}\omega^{2}}$,

$D_{y}=-i\epsilon_{0}E_{z}\epsilon_{d}+\epsilon_{0}\epsilon_{c}E_{y}-\frac{J_{2}}{\mu_{0}\omega^{2}}-\epsilon_{0}E_{y}\epsilon_{s}$,

$D_{z}=\epsilon_{0}\epsilon_{c}E_{z}+\epsilon_{0}\omega_{p}^{2}\epsilon_{d}(iE_{y}-E_{z}\frac{\omega}{\omega_{1}})$,

$\boldsymbol{B}=-\frac{1}{i\omega}\nabla\times\boldsymbol{E}$,

$\boldsymbol{H}=\frac{1}{\mu_{0}}\boldsymbol{B}$,

The electromagnetic force calculation is then derived by integrating of Maxwell stress tensor [Eq.(3) in the main text] along a closed surface surrounding the object. The surrounding surface is chosen as the same shape as the material object (sphere, cubic, prism, and ellipsoid) but with a scaled size $1.1\times$. The entire model's meshing is carefully adapted to ensure the convergence and reliability of all outcomes, as shown in Fig. S12.

Note III. Band structure of the sandwiched Weyl-air-Weyl (WAW) structure

As the dispersive and nonlocal effective parameters is assumed for the minimal ideal Weyl medium in this work, a general numerical software (such as CST and COMSOL) cannot be applied to solve the corresponding eigen value problem.

Hence, a k-form method is adopted to find the localized interface modes in the sandwiched structure, which is implemented as following: Firstly, we rewrite the weak form provided in the last section by using the rule: $\partial_{i}A\to\partial_{i}\tilde{A}-ik_{i}\tilde{A}$ and $\partial_{i}T\to\partial_{i}\tilde{T}+ik_{i}\tilde{T}$, where $A$ represents the original field function, $T$ is the test function. This is based on the Bloch solution of the electromagnetic field that $A=\tilde{\boldsymbol{A}}\exp\left( -i\boldsymbol{kr} \right)$. Similarly, the weak form of the air layer is also modified. Secondly, the wave vector $\boldsymbol{k}$ is expressed as $\boldsymbol{k=}v\boldsymbol{k}_{\boldsymbol{r}}$, where $\boldsymbol{k}_{\boldsymbol{r}}$ represents a fixed direction such as the $k_{y}$ direction selected in our calculation [Figs. 2(a-c) in the main text]. In this way, a $v$-dependent eigen value problem is constructed for each particular frequency. Finally, the corresponding weak forms are implemented by using a triangle mesh based finite element method (FEM) program (COMSOL in our work) to find the corresponding eigen states. Figures 2(a-c) in the main text show the calculated band structures (mode 1 and mode 2) obtained by this method.

**Reference**

[1] Y. Liu, G. P. Wang, and S. Zhang, A nonlocal effective medium description of topological weyl metamaterials, Laser Photonics Rev. **15** (2021).

**Figures S1-S12:**

**Fig.S1** Three-dimensional (3D) band structure of the minimal ideal Weyl system. The two Weyl points with opposite chirality are marked by the red and blue dots respectively, which both locate at the same frequency ($25.1GHz$).

**Fig. S2** Phase integral condition for obtaining the Fermi arcs. The reflection phase of the interface wave obtained at the upper and lower air/Weyl boundaries are denoted as $\phi_{i}$, $i$ is different for distinct eigenmodes. This phase term is the same for both the upper and lower air/Weyl interfaces since these Weyl materials have Weyl points with opposite chirality but the same position in momentum space. The propagation phase in the air layer is denoted as $k_{z}d$, where $k_{z}$ is the wave vector along the $z$ direction and $d$is the air-layer thickness.

**
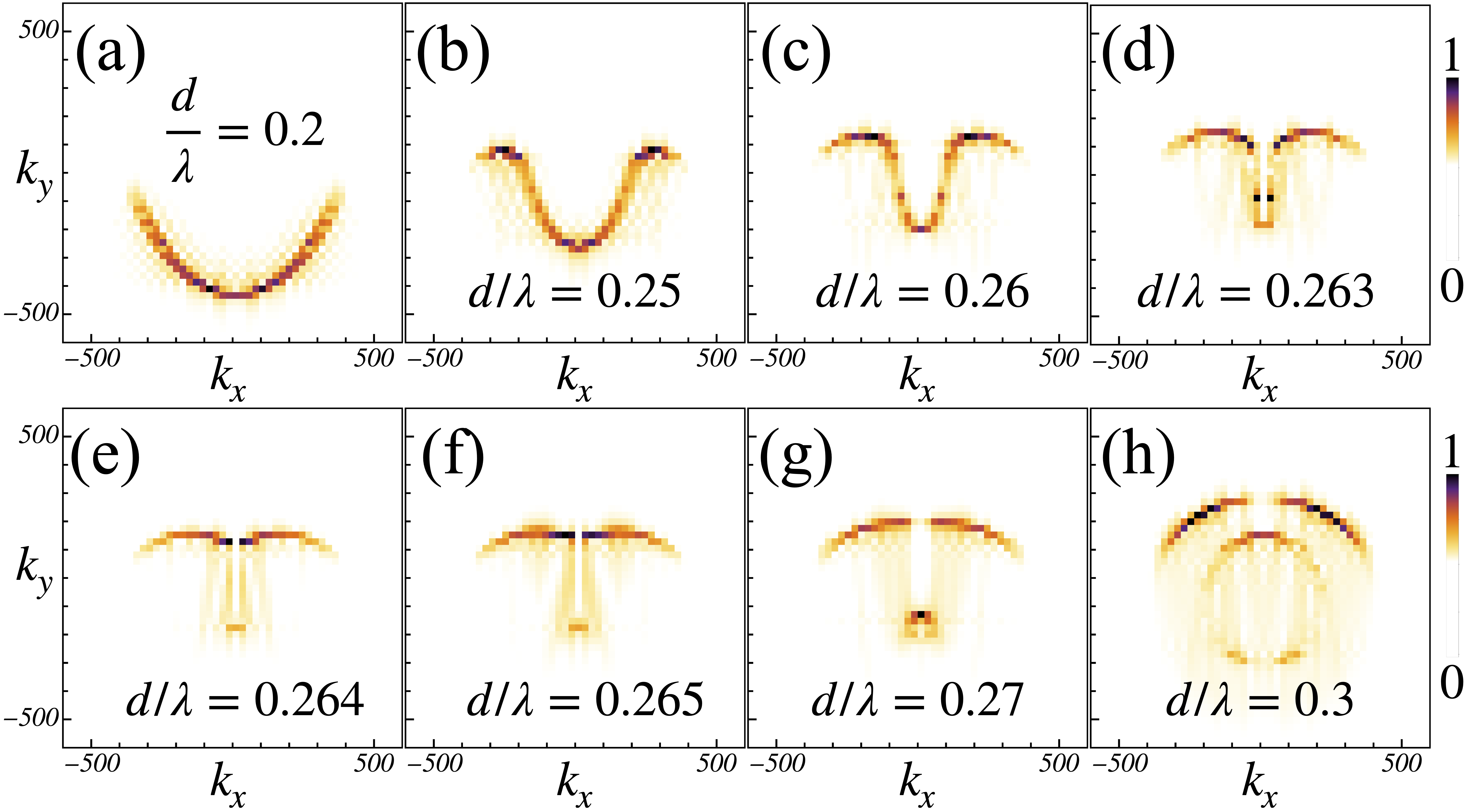
**

**Fig. S3** The geometry of Fermi arcs (mode 1) obtained by the Fourier transformations of the interface waves simulated from full-wave calculations. See Fig. 1 in the main text for theoretical results.

**
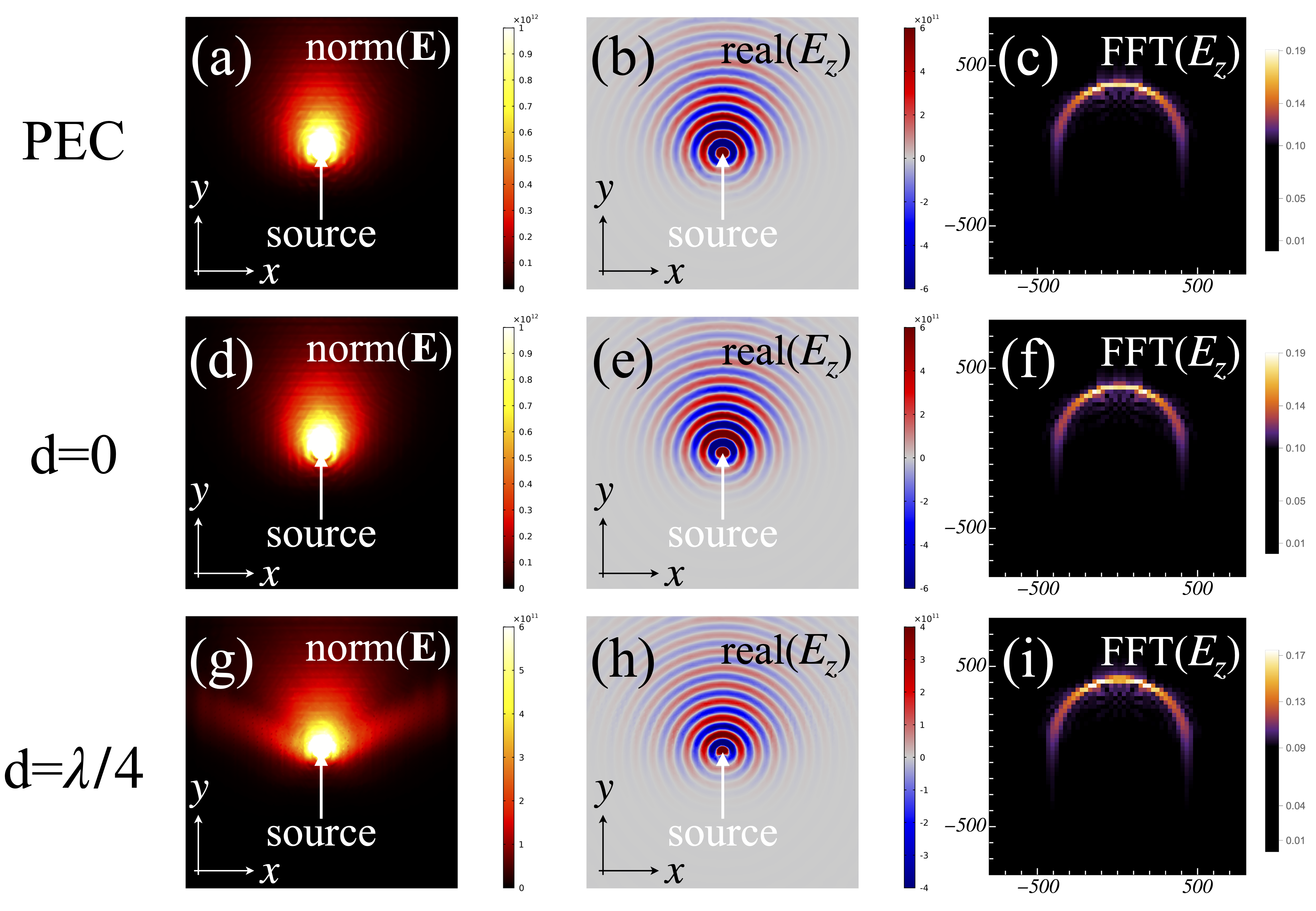
**

**Fig. S4** (a)-(c) The electric fields $norm\left( \boldsymbol{E} \right)$, $real\left( E_{z} \right)$, and $FFT\left( E_{z} \right)$ obtained at the PEC boundary of the minimal ideal Weyl material. (d)-(f) and (g)-(i) are the interface fields obtained at the intervening air layer of the WAW structure, where two different thicknesses $d=0$ and $d=\lambda/4$ are selected respectively. An electric dipole pointing in the z direction is located at the center of the boundary/interface plane to excite the convex Fermi arc waves (mode 2).

**
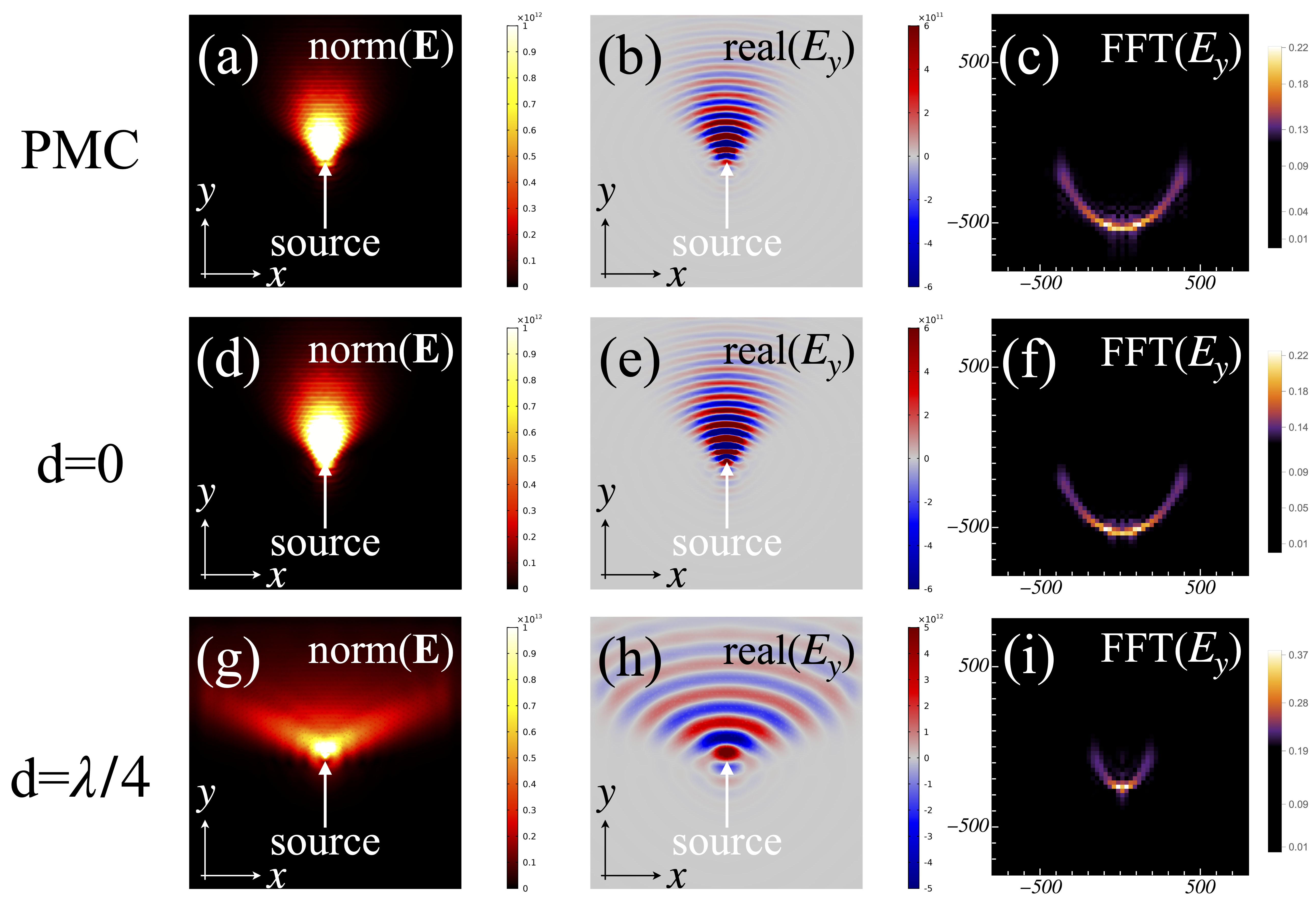
**

**Fig. S5** The concave Fermi arc waves (mode 1) is excited by an electric dipole pointing in the y direction. The plots of $norm\left( \boldsymbol{E} \right)$, $real\left( E_{y} \right)$, and $FFT\left( E_{y} \right)$ are presented correspondingly. (a)-(c) The PMC boundary of the minimal Weyl medium is studied. (d)-(f) and (g)-(i) are the results for the air layers with thicknesses $d=0$ and $d=\lambda/4$ respectively.

**Fig. S6** The momentum conservation law along the $y$ direction. The concave cyan curve shows the Fermi arc of the mode 1 states, where $\boldsymbol{S}$ is the direction of energy flow. The linear momentum of the incident wave in along the $y$ direction (purple arrow) but with a negative value, $\boldsymbol{k}_{in}<0$. Then, it will be scattered to the off-axis direction by the scatterers, $\boldsymbol{k}_{sca}<0$. Due to the magnitude decrease of the negative momentum $\left| \boldsymbol{k}_{sca} \right|<\left| \boldsymbol{k}_{in} \right|$. A negative momentum along the $y$ direction will be imparted to the scattered object $-\Delta\boldsymbol{P}_{y, light}$.


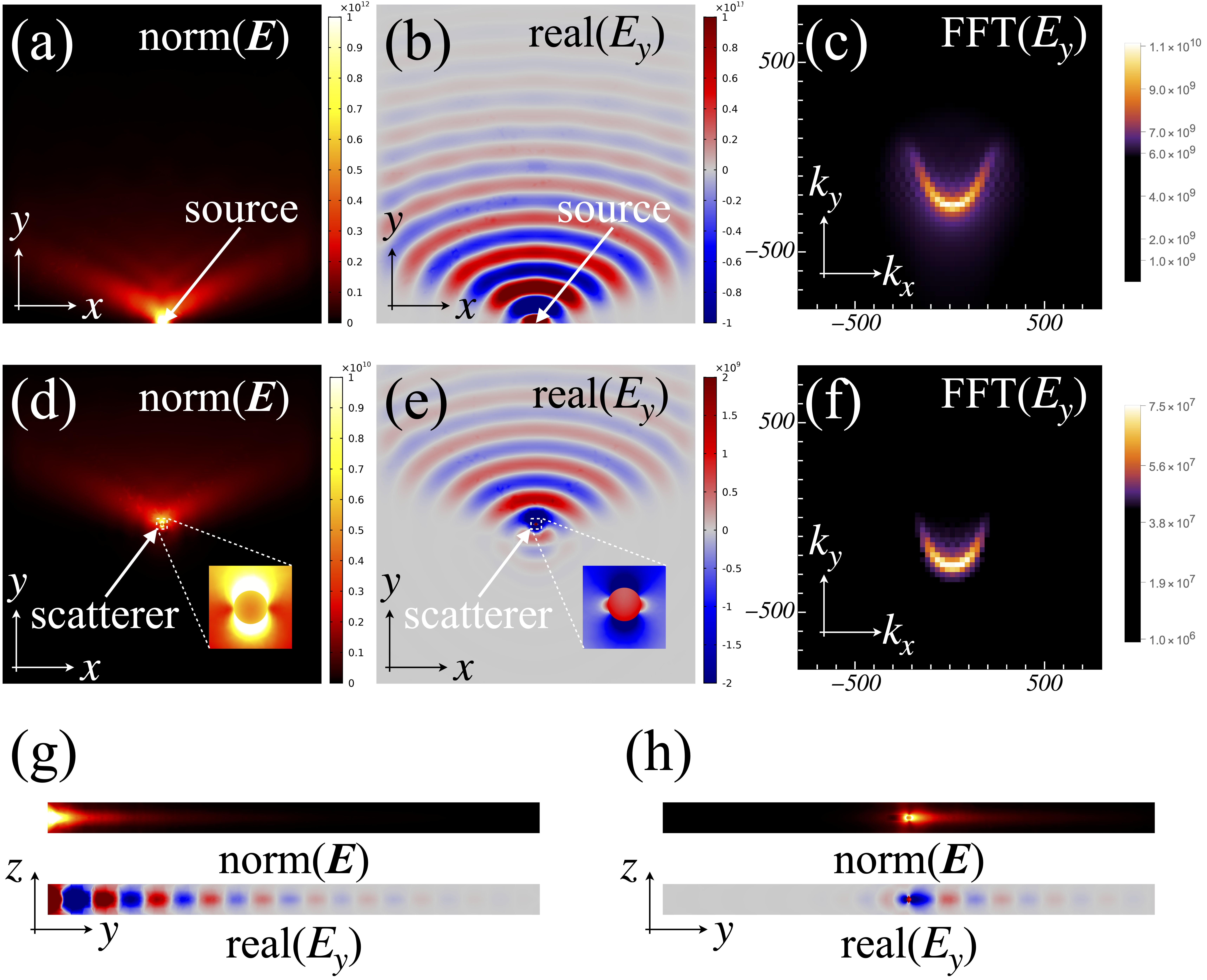


**Fig. S7** The scattered fields obtained when the concave Fermi arc waves encountering a spherical object. (a)-(c) Middle sections of the background $norm(\boldsymbol{E})$, $real(E_{y})$, and $FFT(E_{y})$ fields excited by a source dipole located at the lower edge center of the simulation region. The width of the simulation region is selected as $20\lambda$. (d)-(f) The scattered fields obtained when a spherical scatterer is placed in the air layer (the center of simulation region). Insets show the zoom-in images around the scatterer. (g) and (h) The background and scattered fields in the air region plotted in the y-z plane. For all of these plots, the thickness of the air gap is $d=\lambda/4$, the radius and material permittivity of the spherical scatterer are $\lambda/10$ and $\epsilon=4$. It is evident that the mode of interface field is not changed in the scattering process.


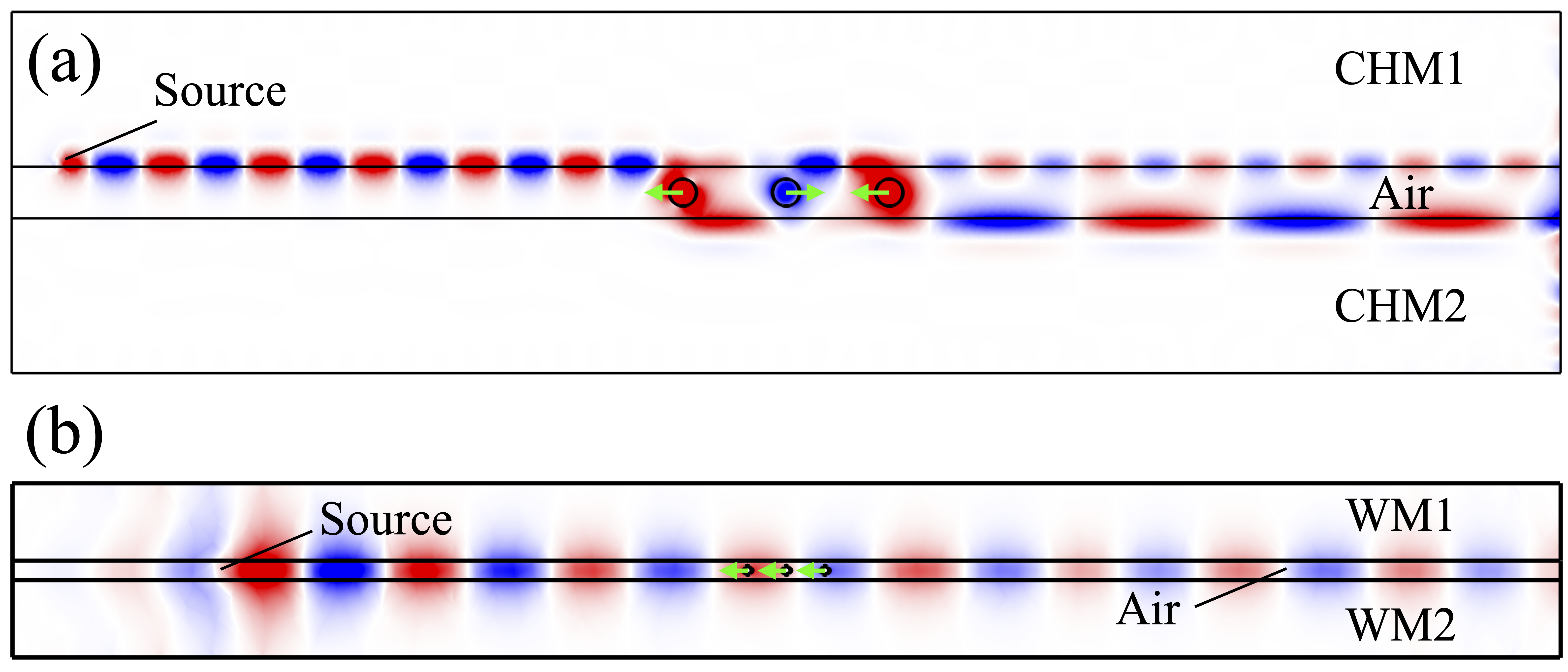


**Fig. S8** Comparison between the two different schemes that use the topologically unidirectional waves to achieve a negative pulling force. (a) An air layer is sandwiched by two chiral hyperbolic materials (CHM) to support the unidirectional interface wave. Three dielectric balls ($\epsilon=4$) are enclosed in the air layer to test the electromagnetic force. Green arrows show the direction of total electromagnetic force obtained by the corresponding balls. Mode-conversion is occurring in this scattering process as the field pattern on the upper and lower interfaces are distinct to each other. The total force exerted on the second ball becomes pushing as the mode-conversion process is reversed. See Ref. [22] in the main text for details of the CHM. (b) In our case, only the concave Fermi-arc waves are excited in the air layer. Thus, the mode-conversion process is nonexistent, which ensures that the electromagnetic force is always pulling for multiple scatterers.


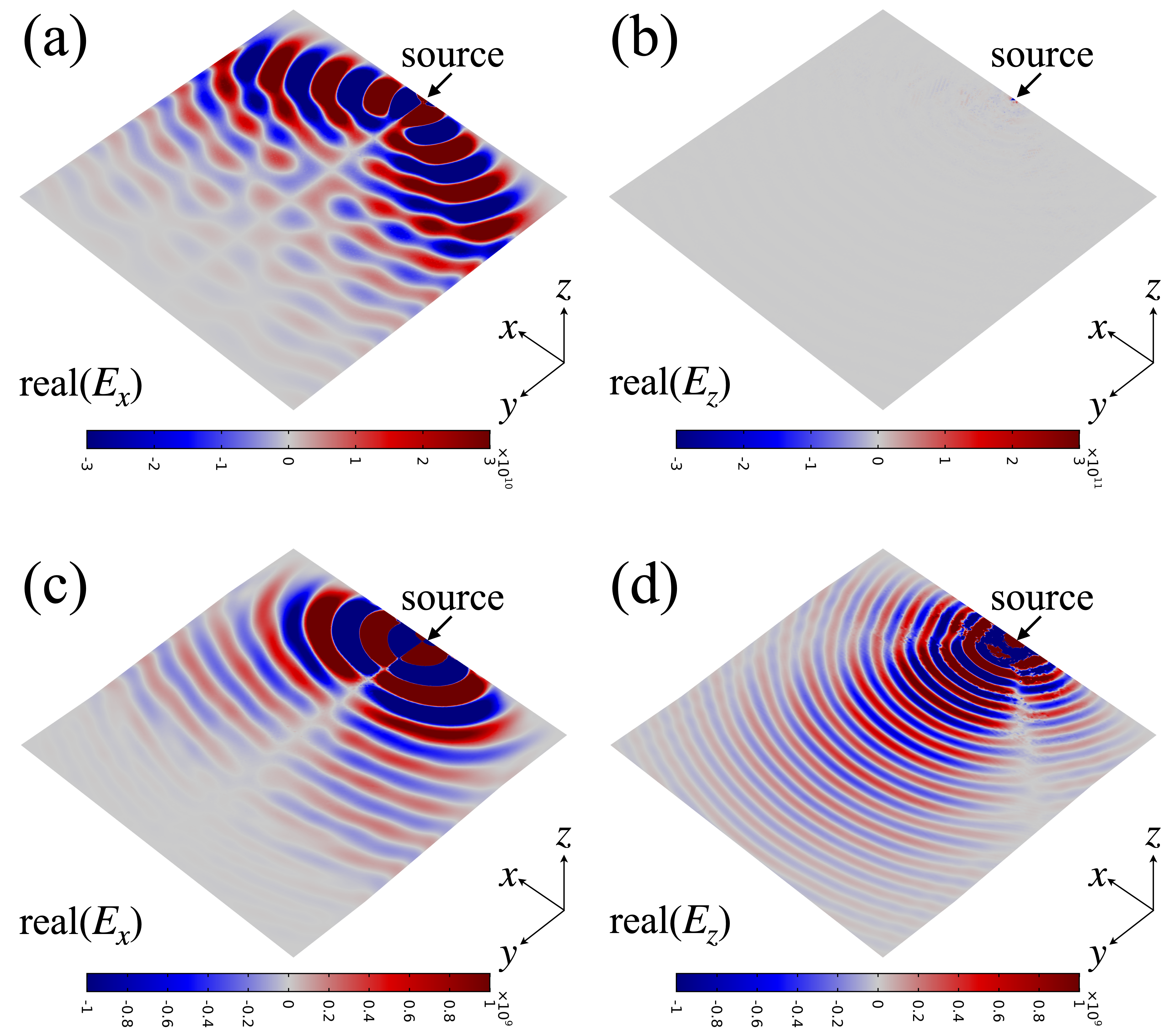


**Fig. S9** (a) and (b) The $E_{x}$ and $E_{z}$ fields obtained at the planar air layer showing in the main text Fig. 3(d). (c) and (d) Similar to (a) and (b) but for the sloping air layer showing in the main text Fig. 3(e).

**
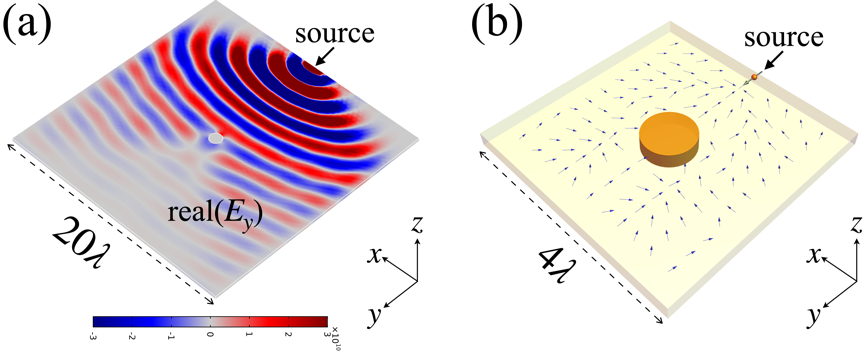
**

**Fig. S10** Distributions of the electric field and force vectors are plotted, respectively, when a cylindrical obstacle made of perfect electric conductor (PEC) is enclosed in the air layer.

**
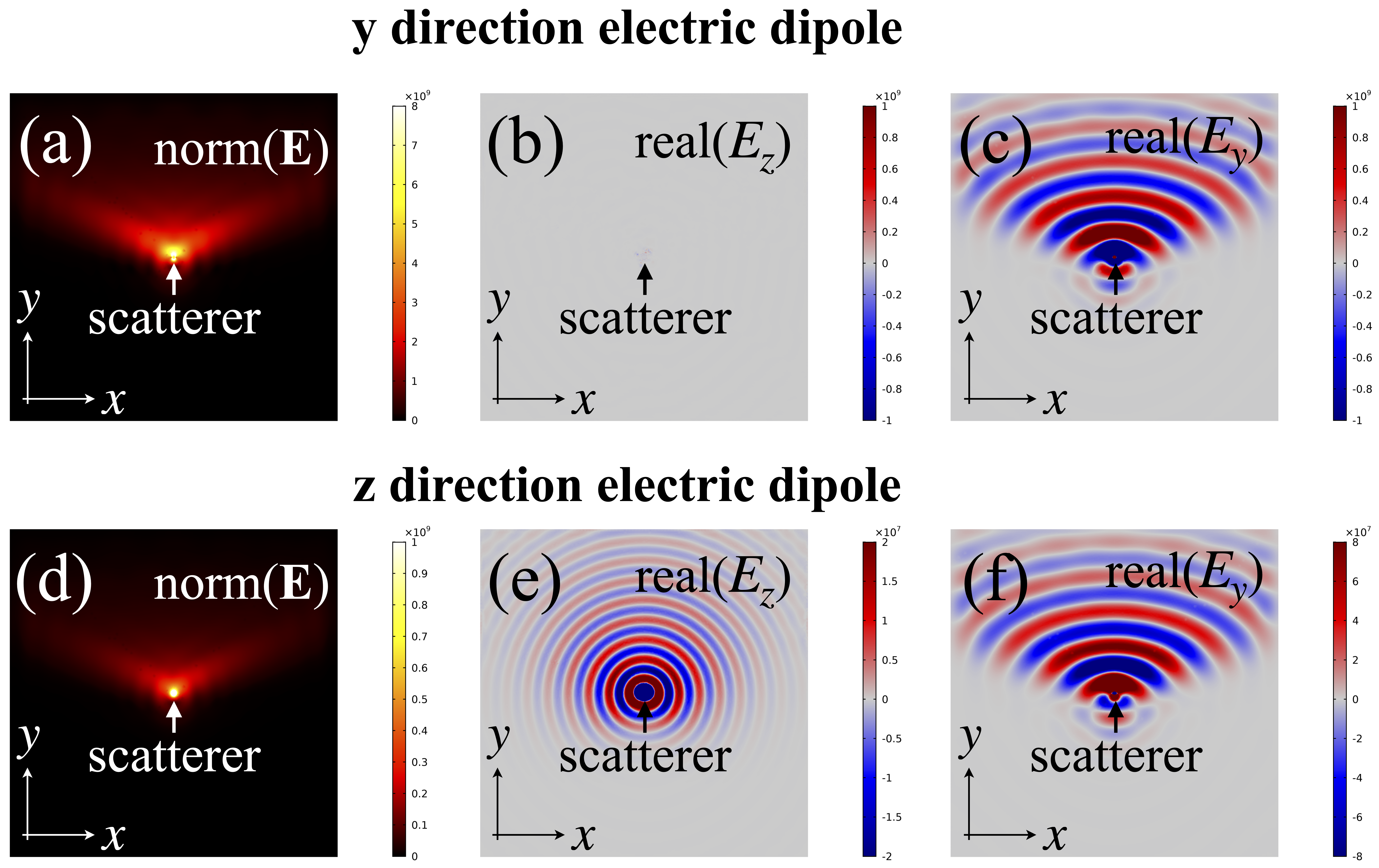
Fig. S11** A spherical scatterer is placed at the center of plane to demonstrated the scattered fields excited by different sources. (a)-(c) An electric dipole polarized in y direction is applied as the source of waves, which is located at the lower edge middle of these figures [same as the configuration presented in Fig. S4(a)]. (d)-(f) Similar to (a)-(c) but for a source dipole polarized in z direction, which shows that a significant part of the scattered field is still a mode 1 state (concave). This part of concave waves contributes to the negative pulling force in this case.


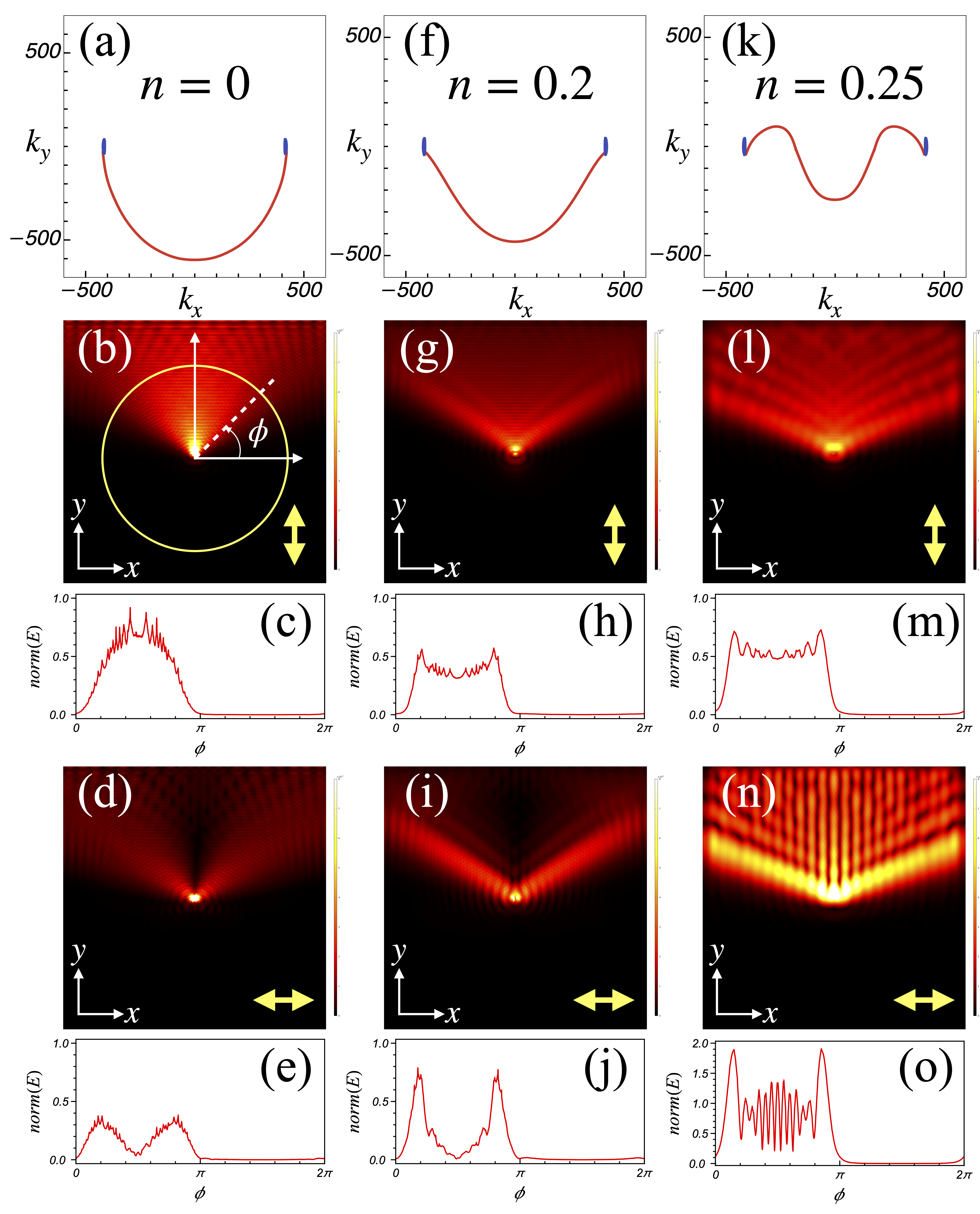


**Fig. S12** Dependence of electric-field strength on the azimuthal angle $\phi$ for different shapes of Fermi arcs. (a) The concave Fermi arc obtained when the air-layer thickness is zero. (b) The electric-field strength distribution resulting from using an electric dipole source along the y-axis. (c) Field strength obtained along the yellow circle shown in (b). (d) and (e) Similar to (b) and (c) but for the dipole source along the x-axis. (f)-(j) and (k)-(o) Similar to (a)-(e) but the Fermi arcs in different shapes (different air-layer thicknesses). It is clear that the shape of Fermi arcs changes the distribution of field strength along the azimuthal angle, which leads to the peaks of force magnitude shown in Fig. 4(f) of the main text.

**Fig. S13** Comparison between our proposal and the general scheme. (a) In our system, the two Weyl points (blue and red dots), which are connected by Fermi arcs (orange curve) in the surface Brillouin zone, are situated at the opposite sides of the original point ($\Gamma$), due to the constraint of the spatial inversion symmetry (the time-reversal symmetry is broken in our system). This suggests that the permitted k vectors (shown by the black dashed arrows) can cover half of the directions within the $k_{x}$-$k_{y}$ plane (represented by the red-colored zone). (b) In contrast, for the general case, the surface states' segments (arcs) extend away from the center ($\Gamma$ point) of the surface Brillouin zone. This limits the surface waves' propagation to certain angles because only a smaller range of k components is permitted.


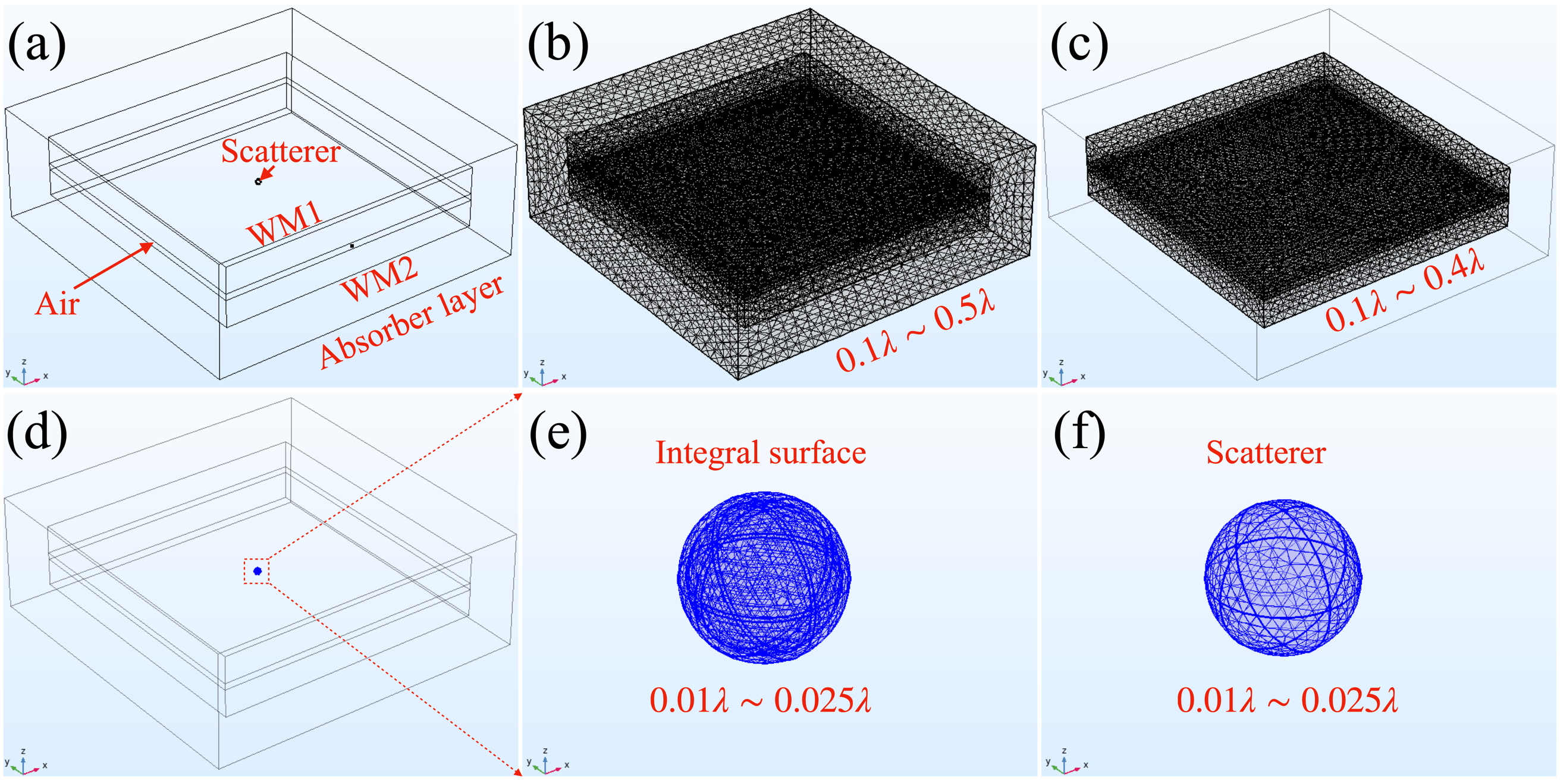
**Fig. S14** Meshing of the WAW model in simulations. The mesh size for each part is indicated in the figures. The entire model is meshed in simulations, and the meshing is carefully adapted to ensure reliable and convergent results.
